# Human iNSC-derived brain organoid model of lysosomal storage disorder in Niemann–Pick disease type C

**DOI:** 10.1038/s41419-020-03262-7

**Published:** 2020-12-12

**Authors:** Seung-Eun Lee, Nari Shin, Myung Geun Kook, Dasom Kong, Nam Gyo Kim, Soon Won Choi, Kyung-Sun Kang

**Affiliations:** Adult Stem Cell Research Center and Research Institute for Veterinary Science, College of Veterinary Medicine, Seoul National University, Seoul, 08826 Republic of Korea

**Keywords:** Disease model, Lipid-storage diseases, Neural stem cells

## Abstract

Recent studies on developing three-dimensional (3D) brain organoids from stem cells have allowed the generation of in vitro models of neural disease and have enabled the screening of drugs because these organoids mimic the complexity of neural tissue. Niemann-Pick disease, type C (NPC) is a neurodegenerative lysosomal storage disorder caused by mutations in the NPC1 or NPC2. The pathological features underlying NPC are characterized by the abnormal accumulation of cholesterol in acidic compartments, including late endosomes and lysosomes. Due to the inaccessibility of brain tissues from human NPC patients, we developed NPC brain organoids with induced neural stem cells from NPC patient-derived fibroblasts. NPC organoids exhibit significantly reduced size and proliferative ability, which are accompanied by accumulation of cholesterol, impairment in neuronal differentiation, and autophagic flux and dysfunction of lysosomes; therefore, NPC organoids can recapitulate the main phenotypes of NPC patients. Furthermore, these pathological phenotypes observed in NPC organoids were reversed by treatment with valproic acid and HPBCD, which are known to be an effective treatment for several neurodegenerative diseases. Our data present patient-specific phenotypes in 3D organoid-based models of NPC and highlight the application of this model to drug screening in vitro.

## Introduction

Niemann–Pick disease type C (NPC) is the rare neurodegenerative disease caused by mutations of NPC1 (~95%) and NPC2 (~5%) that lead to the progressive neurodegeneration of the central nervous system. NPC results from mutations in either NPC1 and NPC2 which are late endosomal/lysosomal membrane protein and soluble lysosomal proteins, respectively^1–3^. Deficits of NPC1 protein result in impairment in regulation of cholesterol efflux and dysfunction of cholesterol homeostasis^3^. NPC patients exhibit progressive neurodegeneration including Purkinje cell death associated with motor impairment, problems with seizures and speech, and early-onset dementia^4,5^. The most prevalent mutation associated with juvenile-onset phenotype is NPC1^I1061T^, which represents 15–20% of all disease alleles^6^. The NPC1^I1061T^ mutation disrupts NPC1 protein trafficking by promoting the ER-mediated degradation of the mutant protein. Previous studies have suggested that the NPC1 mutation is associated with the aberrant accumulation of unesterified cholesterol and sphingolipids, leading to neuronal failure, which contributes to lethality in NPC disease^7,8^. Because neurodegeneration is the main feature of the disease, we previously studied the neurodegenerative mechanisms using NPC1-deficient animal models^9,10^. NPC^−/−^ mice store abundant unesterified cholesterol in the nervous system and exhibit chronic neurodegeneration and eventually a shortened lifespan. In addition to studies using an in vivo model of NPC disease, we also applied an in vitro model system using induced neural stem cells (iNSCs). We previously optimized the generation of iNSCs from various human cell sources, including blood cells, mesenchymal stem cells, and fibroblasts^11–14^. We investigated NPC disease with disease-specific iNSCs from patient-derived fibroblasts using two reprogramming factors, SOX2 and HMGA2 (ref. ^15^). NPC iNSCs exhibited cholesterol accumulation and neurological defects, representing the pathological characteristics of NPC disease. Although modeling NPC disease with iNSCs in two-dimensional (2D) cell culture recapitulates the pathological characteristics of the disease, the analysis of the pathophysiology associated with the neuronal damage in the brain has been challenging due to the limited availability of human brain tissue. In addition, little is known about early brain development in patients with NPC disease because of the inaccessibility of human tissues. Therefore, modeling the human brain is inevitable for the investigation of NPC disorders.

Recent advances in three-dimensional (3D) organoid culture facilitate the probing of human development and the evaluation of therapeutic approaches in systems that are more physiologically relevant than animal and 2D cell culture models. These 3D organoid models recapitulate the complex cellular behaviors of the developing brain, allowing the study of diseases with fundamental neurodevelopmental mechanisms^16,17^. In particular, brain organoids have been studied to develop models of Alzheimer’s disease, Parkinson’s disease, Miller-Dieker syndrome, and frontotemporal dementia by recapitulating the important neuropathological hallmarks found in many neurodegenerative diseases^18–20^. Thus, studies using disease-specific brain organoids provide an understanding of the pathological mechanisms leading to massive neuronal degeneration.

To date, the underlying pathogenic mechanisms of NPC has not been explored using NPC brain organoids. To investigate which pathological phenotypes, such as cholesterol accumulation, affect neurodevelopment in vitro, we generated 3D brain organoids using NPC iNSCs. Indeed, NPC brain organoids showed significantly reduced differentiation with increased cholesterol accumulation. Gene expression profiles showed that NPC brain organoids exhibited the pathological signatures observed in NPC patients. Here, we generated an NPC brain organoid model using human iNSCs harboring NPC1 mutations. To the best of our knowledge, this is the first report that presents the generation of brain organoids with human iNSCs derived from NPC patients.

## Materials and methods

### iNSCs culture

iNSCs were used as reprogrammed cell lines, as described in our previous study. Normal human fibroblasts (GM05659) and NPC patient fibroblasts (GM03123^(NPC1P237S/I1061T)^, GM18453^(NPC1I1061T/I1061T)^) were obtained from Coriell Institute for Medical Research. These cells were maintained in NSC maintenance media containing a 1:1 mixture of ReNcell media (Millipore, Burlington, Massachusetts) and KnockOut DMEM/F-12 supplemented with StemPro NSC SFM Supplement (Gibco/Life Technologies, St. Petersburg, FL, USA) with bFGF (Sigma-Aldrich, Saint-Louis, Missouri) and EGF (Sigma).

### Organoid culture

On day 0 of organoid culture, the iNSCs were dissociated by Accutase (Gibco) to generate single-cell suspensions. In total, 9000 cells were then plated in each well of an ultralow-binding 96-well plate (Corning, NY, USA) in NSC maintenance media. The neurospheres were incubated for 3 days. On day 4 of the protocol, the neurospheres were transferred to droplets of Matrigel (Corning) by pipetting into cold Matrigel on a sheet of Parafilm with small, 3-mm dimples. These droplets were allowed to polymerize at 37 °C and were subsequently removed from the Parafilm and grown in differentiation media containing a 1:1 mixture of DMEM/F12 and Neurobasal Medium with N2 supplement (Gibco), B27 supplement without vitamin A (Gibco), 2-mercaptoethanol, insulin (Sigma), GlutaMAX (Gibco), and MEM-NEAA (Gibco), following the protocol by Lancaster et al.^21^. After 3 days of stationary growth, the tissue droplets were transferred to an orbital shaker containing differentiation media with B27 supplement with vitamin A (Gibco).

### Reverse transcriptase PCR

After 28 days of culture, total RNA was isolated from each organoid using TRIzol (Invitrogen, Carlsbad, USA) following the manufacturer’s suggestions, and cDNA was synthesized using Superscript reverse transcriptase (Invitrogen). Quantitative real-time PCR was performed using the SYBR Green PCR Master Mix (Applied Biosystems, Foster City, USA), and the mRNA expression of each gene was normalized to that of the housekeeping gene GAPDH. The primer sequences of each gene are demonstrated in Supplementary Table 1.

### Western blot analysis

Lysates were prepared from each organoid with Pro-prep protein lysis buffer (Intron Biotechnology Co., Republic of Korea). The protein samples were separated via 8–15% sodium dodecyl sulfate polyacrylamide gel electrophoresis and transferred to nitrocellulose membranes. The membranes were blocked with a 3% bovine serum albumin solution, and the proteins on the membrane were incubated overnight at 4 °C with primary antibodies. The primary antibodies used were TUJ1 (1:1000, Biolegend, 801202), MAP2 (1:500, Merck, MAB3418), NF (1:1000, Cell Signaling, 2836s), ß-actin (1:1000, CST, 4967), cleaved-caspase-3 (1:1000, CST, 9664S), Caspase-3 (1:1000, CST, 9662), LC3 (1:1000, Novus, NB100), and p62 (1:500, BD Bioscience, 610832). The secondary antibodies used were horseradish peroxidase-conjugated antibodies (Invitrogen, Carlsbad, USA; G21040 and G21234). The protein and antibody complexes were detected using an enhanced chemiluminescence detection kit (GE Healthcare Life Science, Buckinghamshire, UK) and analyzed.

### Histology and immunofluorescence

For the histological and immunohistochemical analyses, each sample was fixed with 4% paraformaldehyde overnight at 4 °C. The fixed tissues were washed with phosphate-buffered saline, dehydrated by immersion in an increasing ethanol gradient, embedded in paraffin, and sectioned at a thickness of 7 μm. The sections were stained with hematoxylin and eosin or used for immunohistochemical staining. For immunohistochemistry, the sections were boiled with antigen retrieval citrate buffer (10 mM sodium citrate, pH 6.0) at 85 °C for 10 min and blocked with 5% normal goat serum (Zymed, San Francisco, USA) for 1 h. The sections were stained with specific primary antibodies overnight at 4 °C at the following dilutions: TUJ1 (1:500), NF (1:500), Ki-67 (1:500), MAP2 (1:500), Caspase-3 (1:1000), and cleaved-caspase-3 (1:500). Subsequently, the sections were incubated with Alexa 488- or 594-labeled secondary antibodies (Invitrogen) for 1 h. For counterstaining, the nuclei were stained with DAPI (Zymed Laboratories Inc.) and mounted with DAKO fluorescence mounting medium (Agilent Pathology Solutions). The relative immune-density of each expression after immunostaining was measured using NIH image J software. Randomly chosen regions were selected and images were analyzed.

### Clearing for 3D fluorescence images

The organoids were fixed in 4% paraformaldehyde at 4 °C overnight and processed as described in the protocol of the CytoVista 3D Cell Culture Clearing/Staining Kit (Invitrogen). Following organoid clearing, the samples were stained with a number of different primary antibodies: TUJ1 (1:200) and SOX2 (1:200). For filipin staining, 50 μg/ml filipin (Cayman, Ann Arbor, MI, USA) was added in a permeabilization buffer at 37 °C for 1 h. For microscopic imaging and analysis, the CellInsight CX7 High-Content Screening platform (Thermo Scientific) was used.

### Cholesterol assay

Five organoids from both WT and NPC cultures, with an average weight of 10 mg, were selected for sample preparation. The organoids were extracted with 200 µl of cholesterol lysis buffer (chloroform:isopropanol:NP-40 (7:11:0.1)) in a micro-homogenizer. After centrifuging the extract for 5 min at 15,000 × *g*, the samples were incubated for 5 h in a 50 °C heat block to remove the chloroform. Then, the dried lipids were dissolved in 200 µl of Cholesterol Assay Buffer by sonicating until homogeneous. To prepare the standard curve, the cholesterol assay kit (Bio Vision, Milpitas, CA) was used to dilute the cholesterol standard. After mixing the reaction reagents, they were incubated for 1 h at 37 °C in the dark. Then, the absorbance of each sample was measured at 570 nm in a microplate reader. The operators and investigators were blinded to the sample preparation throughout the process.

### QuantSeq mRNA-sequencing analysis

Three individual organoids were collected from each group. The total RNA was isolated using TRIzol (Invitrogen) following the manufacturer’s protocol. The RNA quality control was performed by a Bioanalyzer 2100 system, and the RNA concentration was assessed using an ND-2000 spectrophotometer (Thermo Inc., DE, USA). For each sample, cDNA library and sequencing were performed using a QuantSeq 3′ mRNA-Seq Library Prep Kit (Lexogen, Inc., Austria) according to the manufacturer’s instructions. High-throughput sequencing was performed as single-end 75, ~10M read sequencing using NextSeq 500 (Illumina, Inc., USA). ExDEGA (eBiogen, South Korea), an Excel-based differentially expressed gene analysis tool, was used to evaluate the differentially expressed genes (DEGs). Gene ontology analysis was performed to determine the association of the gene products in terms of biological processes, KEGG pathways, and molecular functions. The *p* values and false discovery rate (FDR) were calculated and assigned to each gene, and genes with fold change ≥2 and FDR < 0.05 were considered DEGs. The clustering heatmaps were generated using meV software (TM4 Development Group, USA). The National Center for Biotechnology Information Gene Expression Omnibus (GEO) accession number for the RNA-seq data reported in this paper is GSE157676.

### Statistical analysis

The mean values of all the results are expressed as the mean ± SD. The statistical analyses were performed using GraphPad Prism version 5.0 (GraphPad Software, San Diego, CA, USA). Two-tailed Student’s *t*-test or one-way ANOVA followed by Bonferroni’s test for multi-groups were used for the statistical analyses throughout the experiments. Every samples were selected randomly and analyzed. Every result presented as bar charts, the bar represents the mean, and error bars show the standard error of the mean (SEM). Statistical significance is indicated in the figure legends.

## Results

### Generation of brain organoids from iNSCs

**Fig. 1:**
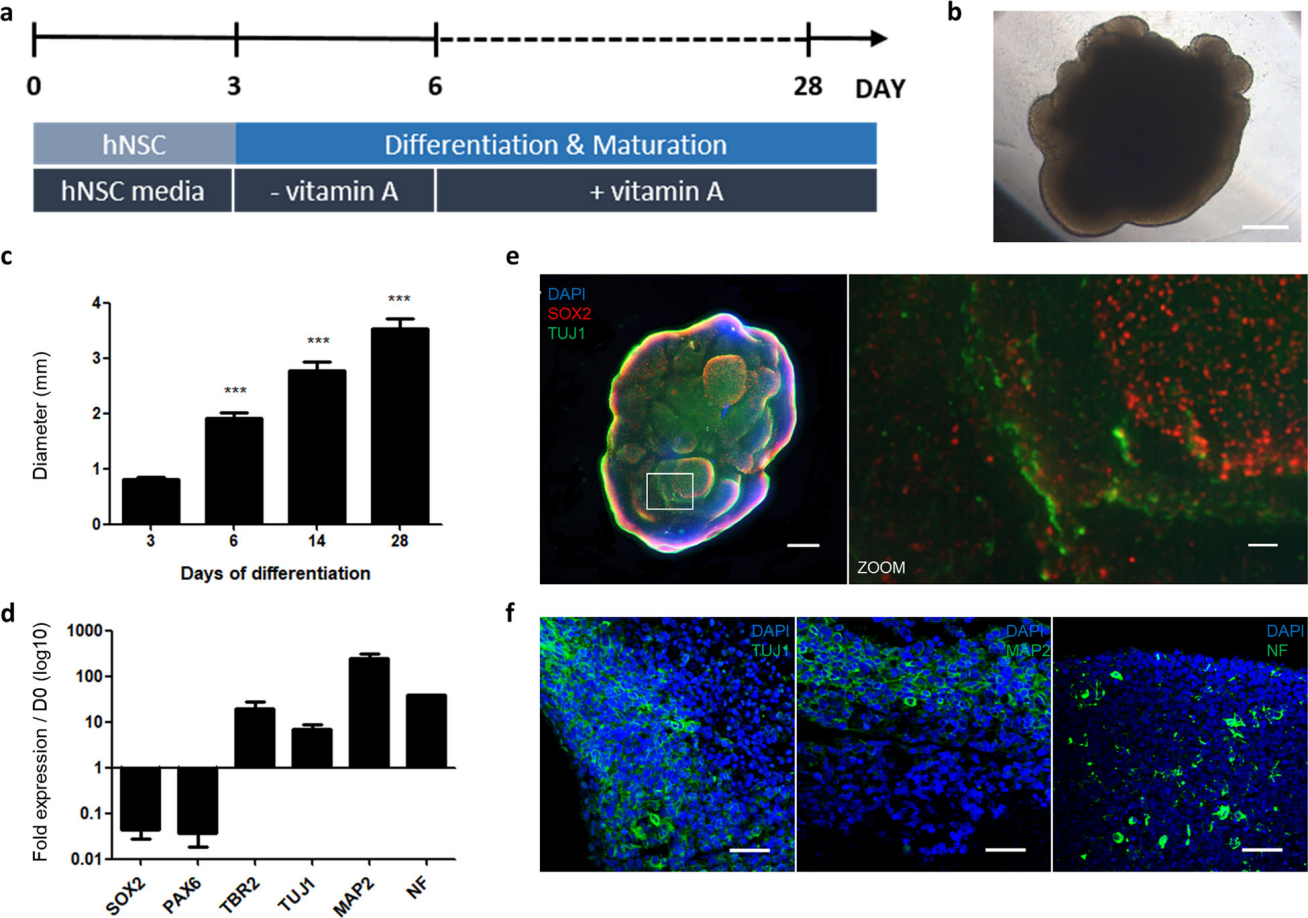
Generation of brain organoids from WT iNSCs. **a** Schematic of the method for the generation of WT organoids using iNSCs. **b** Bright-field images of the organoids at day 28 of maturation. Scale bars, 500 μm. **c** Quantification of the diameter (mm) during the entire course of the brain organoid development under different culture conditions. The results are the mean ± SD. ***p < 0.001. *n* = 10 diameters of organoids were quantified per time point. **d** RT-qPCR analysis of neural progenitor cell markers, *SOX2* and *PAX6*, an intermediate progenitor marker, *TBR2*, and neuron markers, *TUJ1, MAP2*, and *NF*, in the organoids at day 28. The data are normalized to iNSCs at day 0. The results are the mean ± SEM. *n* = 3 per sample. **e** Clearing and immunohistochemistry revealed the whole-mount morphology with heterogeneous regions containing neural progenitors (SOX2, red) and neurons (TUJ1, green). Scale bars, 500 μm. Zoom scale bars, 60 μm. **f** Immunohistochemical staining of serial sections for neuronal markers (TUJ1, MAP2, and NF) at day 28 of differentiation. Scale bars, 50 μm.

We previously established iNSC lines from the fibroblasts of normal donors and NPC patients^11,15^. To study the molecular mechanisms controlling NPC in a 3D environment, we first generated wild-type (WT) brain organoids from normal human iNSCs. We followed the protocol published by Lancaster et al.^21^ with minor modifications and created dense, 3D neural tissues from human iNSC lines. To generate brain organoids, an equal number of dissociated single cells were seeded to form neurospheres (Fig. 1a). After the neurospheres were embedded in Matrigel, they displayed an expanded epithelium at day 6 and markedly increased in size after 28 days (Fig. 1b and Supplementary Fig. S1a). The growth in the diameter of the organoids from day 3 to day 28 was calculated. The average size of the organoids reached over 3 mm (Fig. 1c and Supplementary Fig. S1b). We observed that the mRNA expression of neuronal markers (NF and MAP2) was significantly increased until day 28 (Fig. 1d). In contrast, the expression of neural progenitor markers (SOX2 and PAX6) was markedly decreased under the differentiation conditions beginning at day 6 (Fig. 1d). To better visualize the whole-mount images of the 3D morphology, we performed immunostaining of WT organoids with TUJ1 and SOX2 clearing procedures on day 28 (Fig. 1e). The precise 3D pattern of the structure was visualized in the external part of the brain organoids. Additionally, we also confirmed the expression of the mature neuronal markers TUJ1, NF, and MAP2 in organoid sections by immunostaining at day 28 (Fig. 1f).

### Characterization of NPC brain organoids compared to wild-type organoids

**Fig. 2:**
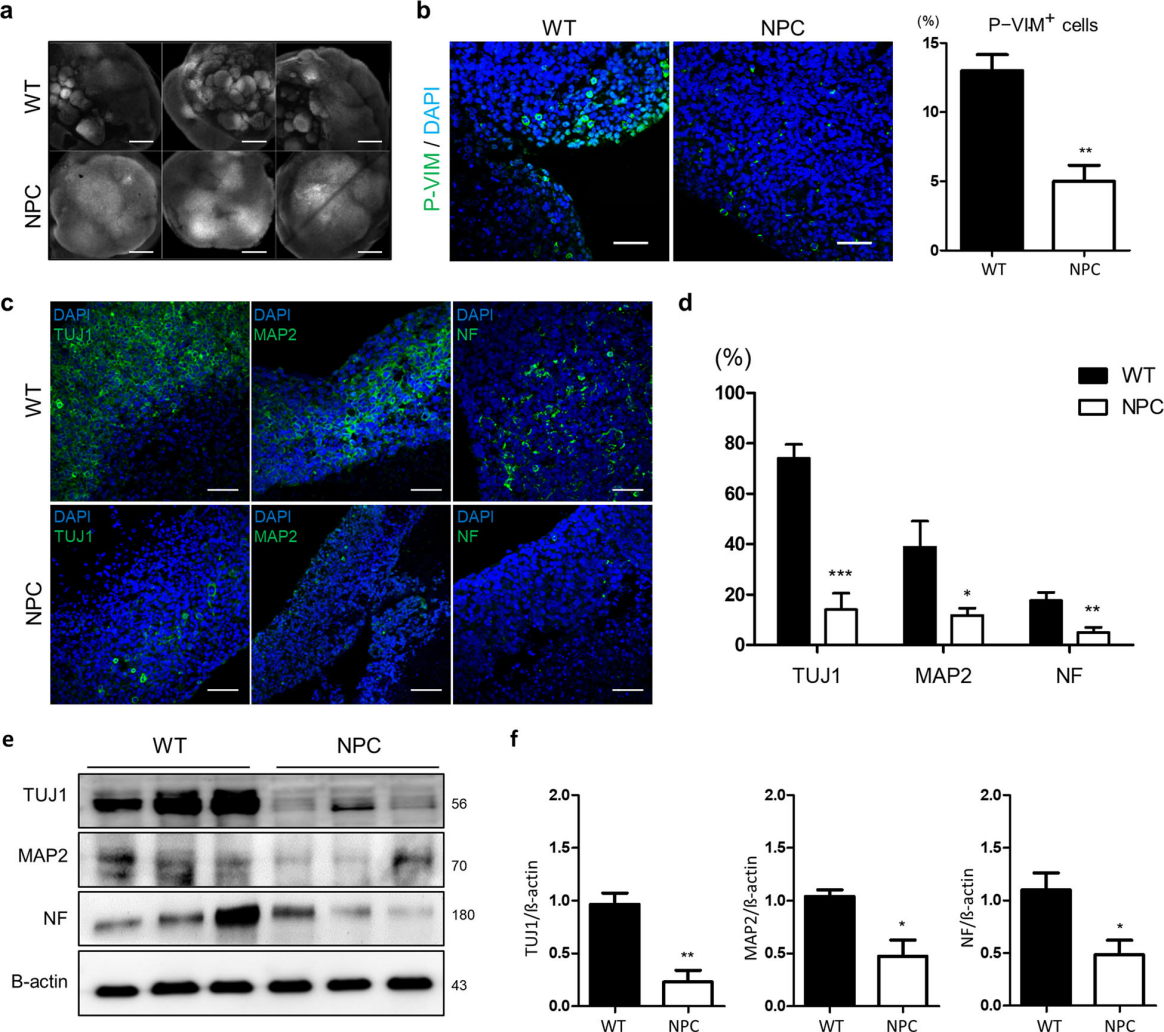
Decreased neuronal markers in NPC organoids. **a** Clearing and immunostaining with DAPI in the WT and NPC organoids at day 28. Scale bars, 500 μm. **b** Immunostaining for mitotic radial glia (phospho-vimentin) in the outer region. Scale bars, 50 μm. **c** Staining for neuronal markers (TUJ1, NF, and MAP2) in the WT and NPC organoids at day 28. Scale bars, 50 μm. **d** The percentage of the total number of cells that expressed TUJ1, NF, and MAP2 of the total number of DAPI-positive cells counted in each group was determined. **e**, **f** Western blot analysis and quantification of neuronal markers (TUJ1, NF, and MAP2) reveals a reduction in the NPC organoids on day 28. Every experiment was performed in triplicate. The results are the mean ± SD. *p < 0.05, **p < 0.01, and ***p < 0.001.

Next, we generated NPC brain organoids following the same procedure used to generate the wild-type organoids. Two NPC patients’ cell lines: GM03123 heterozygous for P237S and I1061T, and GM18453 homozygous for I1061T, were used to generate NPC brain organoids. Compared to the wild-type organoids, the NPC brain organoids at day 28 showed structural and molecular differences. At the early stages of NPC brain organoid generation, the organoids showed delayed formation of the expanded epithelium, and they exhibited a relatively small size after 28 days of culture (Supplementary Fig. S1a, b). NPC brain organoids from each patient, GM03123 and GM18453, exhibited almost the same size and were much smaller than WT organoids. (Supplementary Fig. S1a, b). Next, we also examined the whole-mount 3D images to compare the structures of the wild type and NPC organoids (Fig. 2a). In contrast to the wild-type organoids, the NPC organoids were significantly smaller in size and showed significantly reduced expansion rates. We stained for phospho-vimentin, a marker of mitotic radial glial cells, at day 21 (Fig. 2b). After 28 days, three different neural markers (TUJ1, MAP2, and NF) were expressed specifically in the outer region of the wild-type and NPC organoids using both GM03123 and GM18453 lines (Fig. 2c and Supplementary Fig. S2a). The NPC brain organoids displayed inhibited neural network formation, as indicated by the distribution of neural markers throughout the organoids. Accordingly, immunostaining analysis indicated that the number of cells positive for neural markers was remarkably reduced in the NPC organoids. We observed significant decreases in the proportions of TUJ1-positive cells, NF-positive cells and MAP2-positive cells (Fig. 2d). To estimate whether neural differentiation is impaired at the protein level in the whole NPC organoids, we evaluated the expression of neuronal markers (NF, TUJ1 and MAP2). Western blot analysis revealed that the NPC organoids exhibited substantially decreased levels of neuronal markers compared with the wild-type organoids at day 28 (Fig. 2e, f). Altogether, the NPC brain organoids were characterized by a small size and by reduced neuronal differentiation.

### Limited expansion ability of the NPC brain organoids

**Fig. 3:**
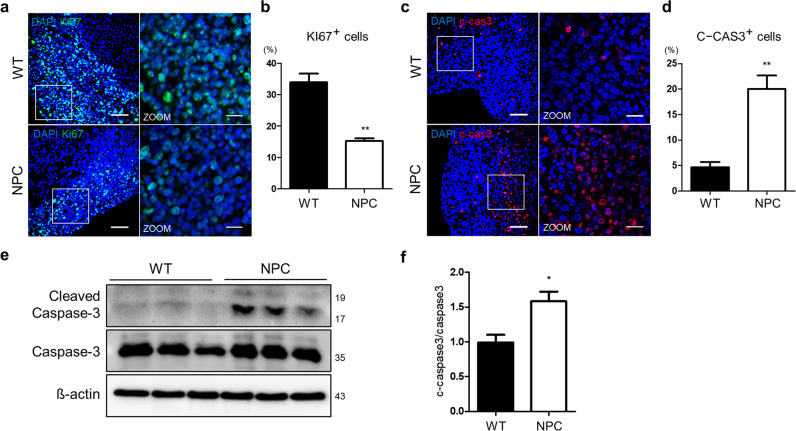
NPC organoids showed decreased proliferation. Comparison of cellular proliferation and apoptosis in the WT and NPC diseased organoids at day 28. **a** Immunostaining for cell proliferation in the WT and NPC organoids on day 28 using antibodies against Ki-67. Scale bars, 50 μm. Zoom scale bars, 10 μm. **b** The quantification of Ki-67-positive cells was conducted following the same method used in Fig. 2d. **c** The WT and NPC organoids were stained on day 28 for the apoptosis marker cleaved-caspase-3. Scale bars, 50 μm. Zoom scale bars, 10 μm. **d** The percentage of cleaved-caspase-3-positive cells was upregulated in the NPC organoids compared with the WT organoids. **e** Western blot analysis and **f** quantification of cleaved-caspase-3 revealed an increase in the NPC disease organoids at day 28. *n* = 3 per group. The results are the mean ± SD. *n* = 3 per group. *p < 0.05, **p < 0.01, and ***p < 0.001.

The regulation of neural progenitor proliferation may contribute to the development of human-specific cortical pattern formation^22^. To demonstrate that NPC brain organoids have limited growth potential, we examined the proliferative ability of the cells inside the organoids to determine the reason for the size difference between the WT and NPC organoids. We investigated the expression of the nuclear proliferation marker Ki-67 in the NPC brain organoids on day 28 (Fig. 3a). The NPC organoids showed a significant decrease in the number of Ki-67-positive cells compared with the wild-type organoids, from 35 to 15% (Fig. 3b). We also found that the apoptotic indicator cleaved-caspase-3 was abundant in the NPC organoids, suggesting that progressive cell death in the neuronal population is a clear pathological hallmark of many neurodegenerative diseases^23^. The NPC organoids showed increased expression of the cellular apoptotic marker cleaved-caspase-3, which increased from 5 to 20% (Fig. 3c, d). By western blotting, we also confirmed a 1.5-fold increase in the pro-apoptosis marker in three independent NPC organoids (Fig. 3e, f), suggesting that cell death is one of the factors that affects the difference in size between the wild type and NPC organoids.

### Cholesterol accumulation is increased in NPC organoids

**Fig. 4:**
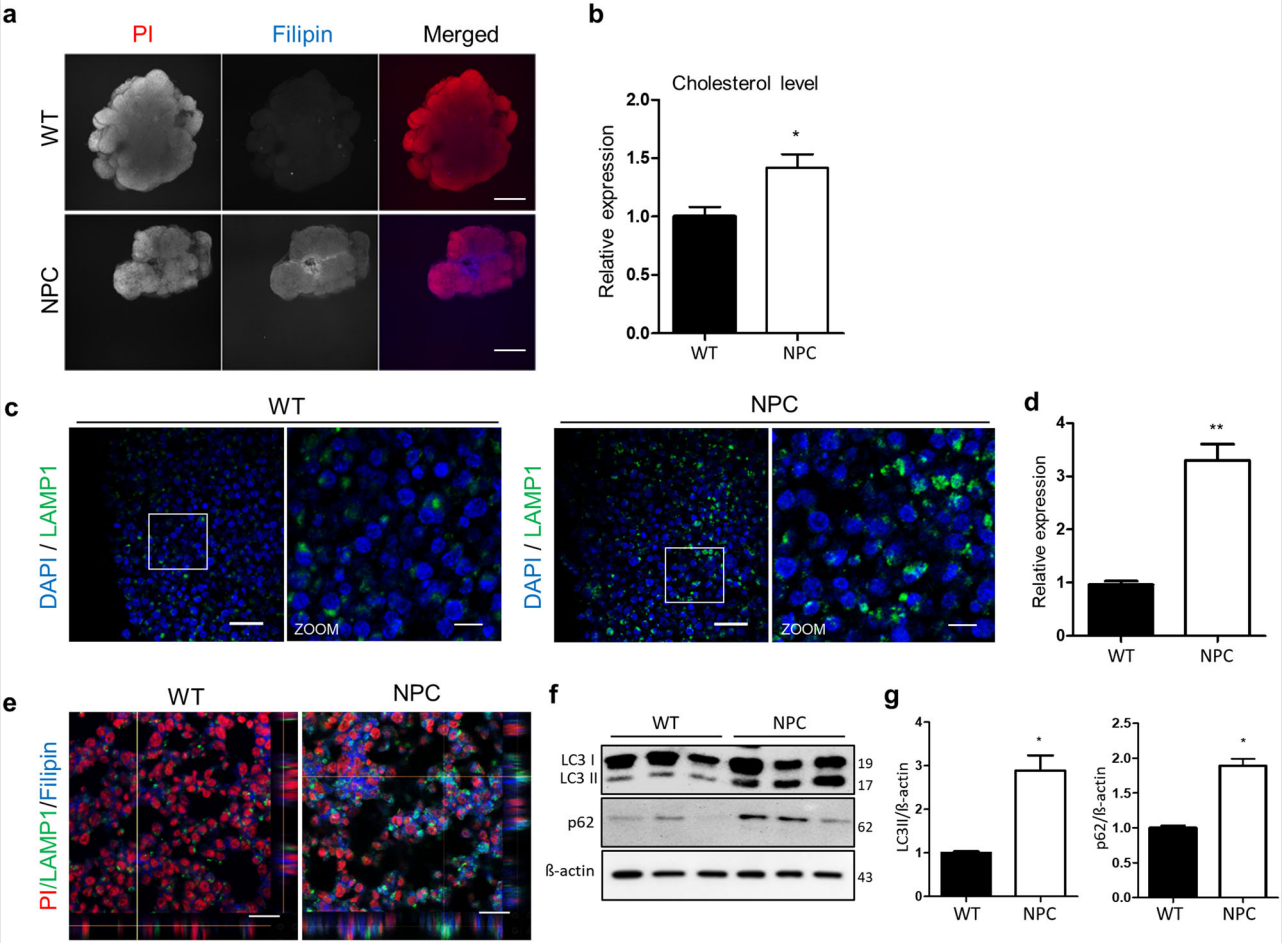
Cholesterol accumulation in NPC organoids. **a** Unesterified cholesterol accumulation was detected by filipin staining (blue) after clearing in the WT and NPC organoids at day 28. Nuclei stained with propidium iodide (red). Scale bars, 500 μm. **b** Cholesterol levels in the WT and NPC organoids were quantified and normalized to those in the WT organoids. *n* = 10 per group. **c** WT and NPC organoids at day 28 were stained for the lysosomal membrane marker LAMP1. Scale bars, 50 μm. Zoom scale bars, 10 μm. **d** The relative number of LAMP1-positive cells was measured for quantification. **e** Co-localization staining of LAMP1 (lysosome marker) and filipin staining through *z*-stack confocal imaging. Scale bars, 20 μm. **f**, **g** WT and NPC organoids were lysed and subjected to western blotting with LC3, p62 antibodies. *n* = 3 per group. The results are the mean ± SD. *p < 0.05 and **p < 0.01.

Dysfunction in the NPC1 protein disrupts cholesterol efflux from the lysosome^24^. Thus, it is suggested that unesterified cholesterol accumulates in the endo-lysosomal system of NPC1-mutant cells^25^. To demonstrate whether unesterified cholesterol accumulation occurs in NPC organoids, we examined the level of cholesterol by filipin staining, which has been widely used for NPC diagnosis^26^. The whole-mount filipin staining of the NPC organoids was conducted with a clearing procedure at day 28 of culture (Fig. 4a). To quantify unesterified cholesterol accumulation in organoids, we selected ten brain organoids of similar weights from each group. Then, cholesterol accumulation in the WT and NPC organoids was quantified using a cholesterol assay kit (Fig. 4b). The relative levels of unesterified cholesterol in the individual NPC organoids were 1.5-fold higher than those in the wild-type organoids. Lysosome-associated membrane protein 1 (LAMP1) has been shown to regulate the stability of the lysosomal membrane^27^. The NPC organoids contained LAMP1 puncta and exhibited a three-fold increase in the number of LAMP1-positive cells, indicating enlarged lysosomes (Fig. 4c, d). Mutations of NPC1 results in the accumulation of unesterified cholesterol within late endosomes and lysosomes^24^. The level of unesterified cholesterol accumulation in lysosomal membrane was indicated by the co-localization of filipin and LAMP1 staining using *z*-stack confocal imaging (Fig. 4e and Supplementary Fig. S3a). In addition to the dysfunction of the NPC1 proteins, which leads to lysosomal accumulation of unesterified cholesterol, the pathological state of lysosomal storage diseases contributes to impaired autophagy^28^. The lysosomal accumulation of unesterified cholesterol inhibits the autophagic flux and inhibits the fusion of autophagosomes (LC3) and lysosomes^29^. Disruption of autophagy was measured by the level of autophagosomes, which correlates with the autophagosome associated form of microtubule associated protein 1 light-chain 3 (LC3-II). To show the inhibition of autophagy in the NPC organoids, both the autophagy markers LC3-II (autophagosome) and p62 (autophagy substrates) were examined in the NPC organoids (Fig. 4f). LC3-II and p62 were increased by 2.5- and 1.5-fold, respectively (Fig. 4g).

### Gene expression analysis of WT and NPC organoids

**Fig. 5:**
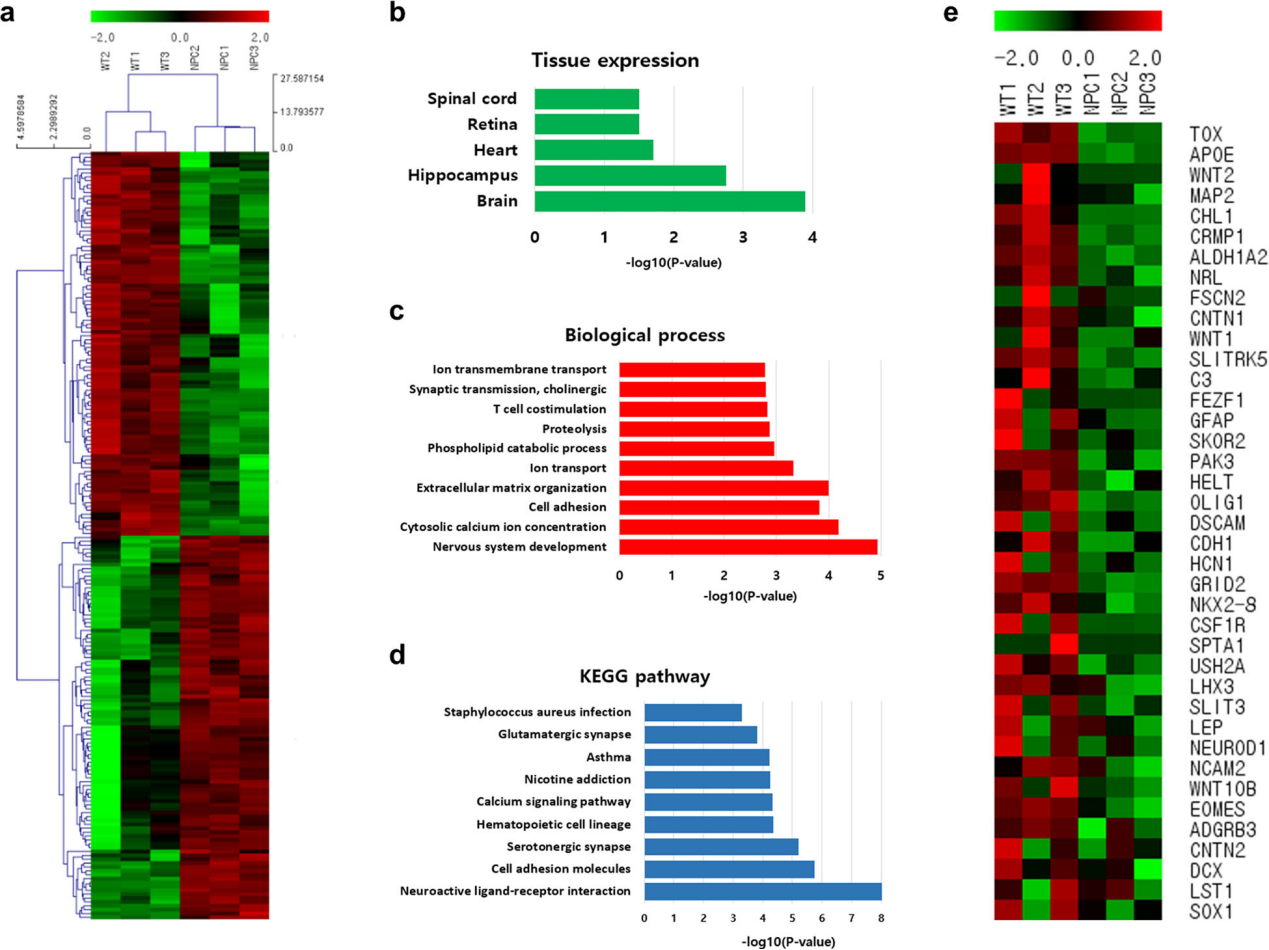
Gene expression analysis of WT organoids and NPC disease organoids. **a** Differentially expressed genes in the WT and NPC organoids at day 28. Heat maps of the *z*-score values of RNA-Seq in the WT and NPC organoids on day 28. Three samples were prepared from each group. **b** Top five tissue with enriched expression of the top 1000 genes that were upregulated in the WT organoids compared with the NPC organoids at day 28. **c** The top 10 highest ranked biological process categories were plotted according to *p* value. **d** KEGG pathways were plotted by selecting the top nine terms ranked according to *p* value. **e** Top 20 genes that were significantly upregulated in the WT organoids compared with the NPC organoids at day 28, presented as a heat map of *z*-score values. Three samples from each group are shown.

We next analyzed whole-transcriptome RNA sequencing to identify the differences in the gene expression profiles of the NPC disease and WT brain organoids. First, we identified a total of 1000 genes that were differentially expressed in the NPC brain organoids relative to the wild-type organoids at day 28. After 28 days of culture, there was a divergence between the NPC and wild-type organoids, suggesting differences in their gene expression profiles (Fig. 5a). A total of 1000 genes quantified in all three samples were analyzed for gene ontology using the DAVID bioinformatics resources tool. The gene ontology analysis of the top 500 genes that were upregulated in the wild-type organoids compared with the NPC disease organoids revealed that the top tissue expression profiles identified were predominantly associated with the brain (Fig. 5b). We further analyzed the biological processes of the differentially expressed genes by mapping through DAVID analysis. Most of the genes in the data set mapped to nervous system development, indicating that most of the genes are relevant to neuronal mechanisms (Fig. 5c). Furthermore, most genes in the KEGG pathway were related to neuroactive ligand-receptor interactions (Fig. 5d). Moreover, the top 20 genes among the 100 genes that were upregulated in the wild-type organoids compared to the NPC disease organoids encoded transcription factors, including *NTF8, FES, MAP2, CHL1*, and *TAL1* (Fig. 5e). The elevated expression of these genes showed that neuronal differentiation was increased in the wild-type organoids than in the NPC organoids.

### Valproic acid treatment increases the number of neuronal-positive cells in NPC organoids

**Fig. 6:**
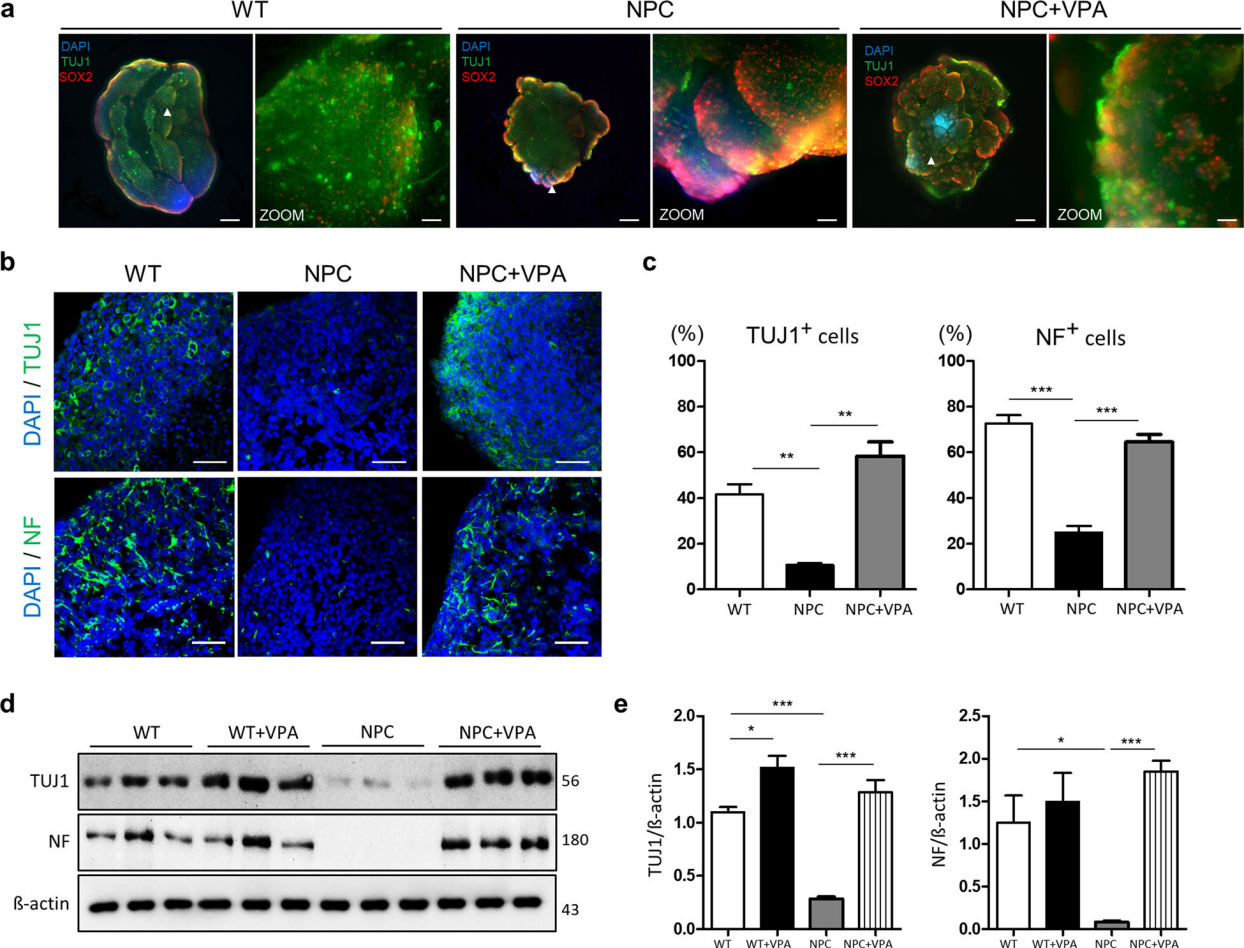
VPA rescues neuronal differentiation in NPC organoids. **a** Clearing and immunostaining for TUJ1 and SOX2 in the NPC organoids after treatment with 1 mM VPA for 1 week. Scale bars, 500 μm. Zoom scale bars, 60 μm. **b** Immunostaining for neuronal markers (TUJ1 and NF) in the sections from the NPC organoids and VPA-treated organoids on day 28. Scale bars, 50 μm. Zoom scale bars, 10 μm. **c** Quantification of the expression of each marker was performed following the same method used in Fig. 2d. **d**, **e** Western blot analysis and quantification of TUJ1 and NF. *n* = 3 per group. The results are the mean ± SD. *p < 0.05, **p < 0.01, and ***p < 0.001.

Next, we investigated ability of pharmacological treatment to rescue the NPC pathological phenotypes in the 3D brain organoid cultures. We focused on treatment with one of the HDAC inhibitors, valproic acid (VPA), which is one of the best-known HDAC inhibitors in several clinical trials. We already described the therapeutic effects of VPA in the NSCs of a mouse model of NPC^9^. To evaluate the preventive effect of VPA on the NPC organoids, VPA was administered for a week starting at 3 weeks of culture. At day 28, we assessed the morphological and structural changes by 3D confocal imaging. The VPA-treated organoids exhibited significantly increased size and pattern formation of the outer layer compared to the non-treated NPC organoids (Fig. 6a). After treatment with VPA, the level of TUJ1-expressing cells increased and a similar number of TUJ1-positive neurons was observed in the VPA-treated NPC organoids compared to the wild-type organoids. To examine the rescue of neuronal differentiation, we investigated the changes in the neuronal marker (TUJ1 and NF) expression pattern after 7 days of VPA treatment (Fig. 6b). At day 28, the VPA-treated NPC brain organoids exhibited significantly upregulated in the neuronal (TUJ1 and NF) expression compared with the non-treated NPC organoids. TUJ1 and NF expression of VPA-treated organoids were detected in approximately 70% of the DAPI-stained cells. The percentage of TUJ1 and NF-expressing cells was significantly decreased to 10% and, 20% in the NPC brain organoids, respectively (Fig. 6c). Furthermore, we also tested HPBCD, a drug that is well-known as a cholesterol transporter to reduce cholesterol accumulation and is effective in prolonging survival in NPC disease animal model^30^. HPBCD was effective in neuronal differentiation in NPC brain organoids using both GM03123 and GM18453 patients’ cells (Supplementary Fig. S4a–d). The effectiveness of both drugs, VPA and HPBCD, is similar to neuronal differentiation in NPC brain organoids. Additionally, we performed western blotting on whole-brain organoid lysates at day 28 to investigate the neuronal (TUJ1, MAP2, and NF) expression at the cellular level after VPA treatment of the NPC brain organoids. Interestingly, the VPA-treated NPC organoids significantly upregulated the expression level of TUJ1 compared to the nontreated NPC organoids and the WT organoids. Additionally, we performed western blotting on whole-brain organoid lysates at day 28 to investigate the neuronal marker (TUJ1 and NF) expression at the cellular level. Similar to the previous immunostaining results, the expression level of both neuronal markers was dramatically decreased in NPC brain organoids, and significantly reversed after VPA treatment (Fig. 6d–e). To better identify the genes throughout the genome that are regulated by VPA treatment, we performed RNA sequencing of the VPA-treated and nontreated NPC organoids. We identified the top five biological processes associated with 1000 upregulated genes in the VPA-treated NPC organoids: regulation of cytosolic calcium ion concentration, axon guidance, cell–cell signaling, nervous system development, and immune system (Supplementary Fig. S5a). The molecular process analysis showed that the top five pathways associated with 1000 upregulated genes in the VPA-treated NPC organoids were calcium ion binding, growth factor activity, neuropeptide hormone activity, hormone activity, and cytokine activity (Supplementary Fig. S5b). Additionally, the clustering of 50 genes related to neuronal function demonstrated significant differences between the VPA-treated and nontreated groups (Supplementary Fig. S5c). The expression of these genes in the VPA-treated NPC organoids was significantly rescued and was similar to that in the WT organoids. Therefore, we assumed that VPA potentially affects neuronal function in NPC.

### VPA rescues NPC organoids via activation of autophagic signaling

**Fig. 7:**
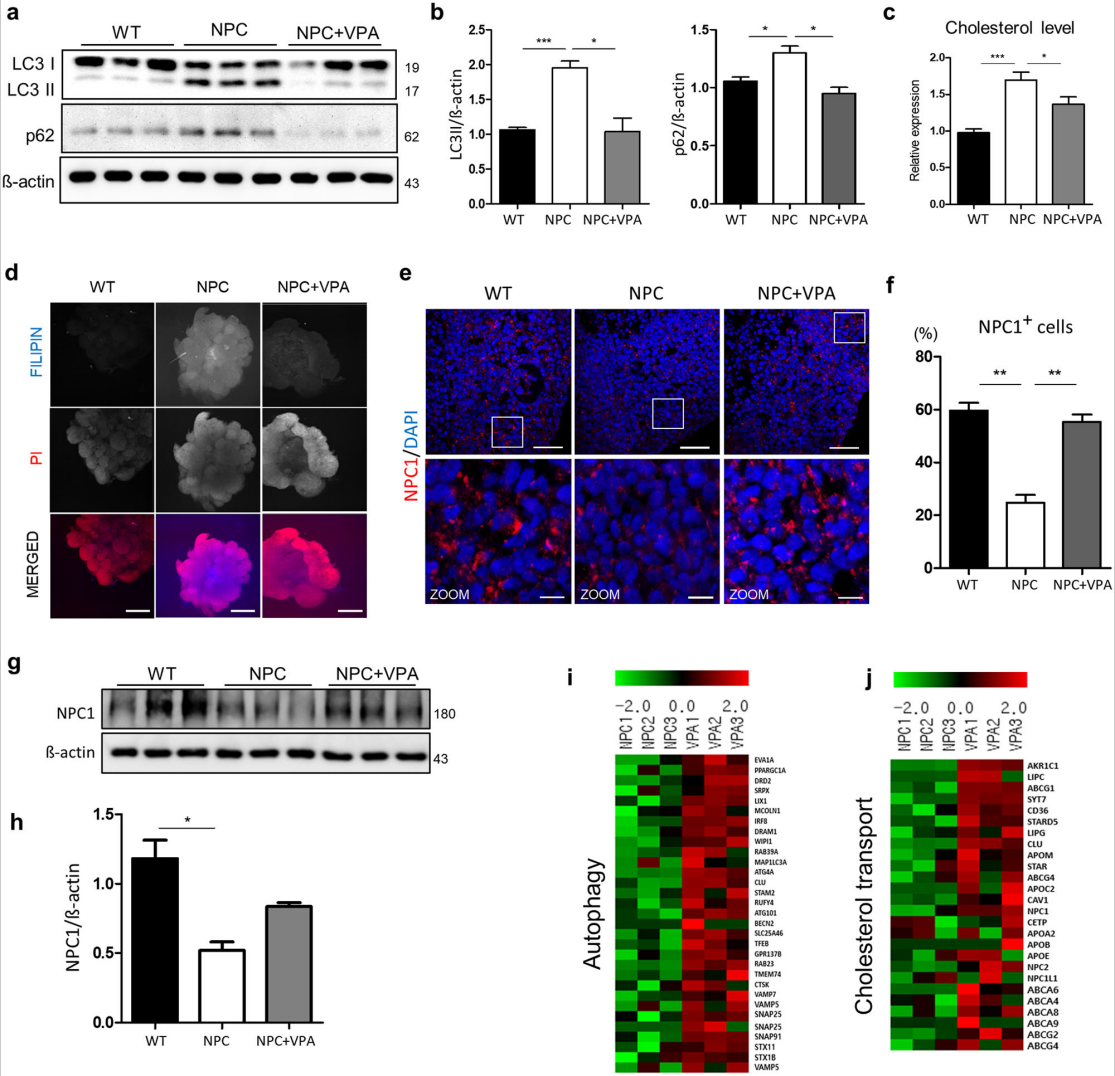
The therapeutic effect of VPA is dependent on autophagic signaling. **a** Western blot analysis and **b** quantification of the LC3-II and p62 levels in the WT, NPC, and VPA-treated NPC organoids. *n* = 3 per group. **c** Cholesterol levels in each organoid were quantified and normalized to those in the WT organoids. *n* = 10 per group. **d** Whole-mount filipin staining of the WT, NPC, and VPA-treated NPC organoids on day 28. Scale bars, 500 μm. **e** Immunostaining for NPC1 in the WT, NPC, and VPA-treated organoids. **f** Quantification of NPC1 staining in each group. Scale bars, 50 μm. Zoom scale bars, 10 μm. **g** Western blot analysis of NPC1 marker and **h** quantification of each group. *n* = 3 per group. **i**, **j** Heat map of the differentially expressed genes related to cholesterol transport and autophagy in the NPC and VPA-treated NPC organoids. *n* = 3 per group. *p < 0.05 and ***p < 0.001. The results are the mean ± SD.

As we mentioned before, impaired autophagic fusion is involved in NPC disease, and cholesterol accumulation and lysosomal damage lead to autophagic stress and eventually to neuronal death^28^. VPA is known to have important therapeutic potential in the treatment of lysosomal storage disease through the enhancement of autophagy. First, to examine whether VPA treatment affects the autophagic flux in NPC organoids, we investigated the levels of LC3-II and p62 in whole organoid lysates by western blot (Fig. 7a). The VPA-treated NPC organoids exhibited decreased expression of LC3-II and p62 compared to the nontreated NPC organoids (Fig. 7b). These results also showed that the autophagic flux was rescued in the VPA-treated NPC organoids and restored to the level of the autophagic flux observed in the WT organoids. To investigate the effects of VPA treatment on autophagy-related genes, we compared the transcriptome profiles of the nontreated and VPA-treated organoids (Fig. 7i). VPA treatment enhanced genes involved in the induction of autophagy (*TFEB, RAB39A*, and *RAB23*) and autophagic fusion (*VAMP7, VAMP8*, and *SNAP25*). Second, to investigate whether this rescued autophagic flux is related to reduced cholesterol levels, the unesterified cholesterol in each organoid was quantified using a cholesterol assay kit (Fig. 7c). We also measured cholesterol levels by filipin staining with a clearing procedure (Fig. 7d). As expected, the accumulation of cholesterol was reduced in the VPA-treated NPC organoids (Fig. 7c, d). Previous reports demonstrated that HDAC inhibitors treatment rescued the NPC1 expression in NPC patient’s fibroblast^31,32^. NPC1 expression in NPC organoids was measured after treatment with VPA (Fig. 7e–h). VPA treatment upregulated the NPC1 expression in nontreated NPC organoids. To examine cholesterol metabolism after VPA treatment, we compared cholesterol transport-related genes in the nontreated and VPA-treated organoids (Fig. 7j). The expression levels of *ABC isoform A2, 4, 6, 8, 9*, members of the cholesterol transport family, were downregulated in the NPC organoids and upregulated in the VPA-treated NPC organoids. Additionally, *NPC1*, which encodes a membrane protein that mediates intracellular cholesterol trafficking, was upregulated in the NPC organoids after VPA treatment. Taken together, these results indicate that the specific NPC-like phenotypes observed in the NPC patient iNSC-derived organoids can be reversed with VPA treatment, suggesting that this model is amenable to the study of cholesterol-relevant mechanisms.

## Discussion

The most prominent phenotype of NPC is abnormal endosomal–lysosomal trafficking in the brain and liver, resulting in the accumulation of several lipids in the lysosomes^33^. NPC disease leads to progressive neurodegeneration from the perinatal period to maturity^8^. The development of an established NPC model system is required for understanding the molecular pathogenesis and for investigating effective drugs for NPC treatment. However, to date, no studies have reported studying NPC with 3D brain organoid models that recapitulate the disease-specific phenotypes and indications observed in humans. Previous studies have suggested that 3D organoid models are suitable for modeling disease pathogenesis in many organs, including the brain, retina, kidney and intestine^34–37^. In particular, a number of brain organoids related to neurodegenerative diseases have been suggested as prominent tools for modeling pathological development, such as microcephaly, Parkinson’s disease, Alzheimer’s disease, and autism^19,38,39^. The goal of our study was to establish a human brain organoid model of NPC for the sake of more thoroughly understanding the pathology and mechanisms in a 3D environment system. Furthermore, we identified defects in cholesterol homeostasis and autophagy, which led to the inhibited neuronal development of NPC in the 3D brain organoid model.

We previously demonstrated that established NPC-iNSCs exhibited several disease-specific phenotypes, including cholesterol accumulation, reduced neuronal differentiation, and self-renewal^15^. To further elucidate the disease-related defects in environments similar to those observed in vivo, this study examined other defects in the NPC brain organoids, including defects in apoptosis and autophagy. Autophagy is important for neurodegenerative diseases, since impaired autophagy causes aggregation formation and affects cellular toxicity. Therefore, autophagy modulation has been suggested as a potentially beneficial treatment in several neurodegenerative diseases. Autophagy also regulates the metabolism of cholesterol, which is important for intracellular trafficking. The impaired autophagic flux in NPC appears due to the inhibited fusion of autophagosomes and lysosomes, which is attributed to cholesterol accumulation in the late endosomal/lysosomal compartments^29^. Brain-specific abrogation of autophagy is associated with neurodegeneration, suggesting a similar pathological effect of defective autophagy in NPC patients. We also identified reduced neuronal differentiation in the NPC organoids in addition to their reduced size. Further studies are needed to elucidate the mechanism by which the abrogation of autophagy contributes to the decreased neuronal differentiation in NPC organoids.

We demonstrated the validity of this organoid as a suitable model for effective drug testing. In our previous animal study, we examined whether VPA could recover defective cholesterol metabolism and upregulate neuronal differentiation in a mouse model of NPC^9^. We tested drug treatment into the brain organoid model with VPA, which has been proven to be effective in neuronal development. Our results further revealed that treatment of NPC organoids with VPA can restore neuronal differentiation following increased autophagosome–lysosome fusion and reduced cholesterol (Fig. 7). To investigate the alterations that occur after VPA treatment, we compared the gene expression profiles of non- and VPA-treated NPC organoids. Transcript profiling demonstrated that factors involved in neuronal differentiation and neural development *(NFASC, NGF, MAP2*, and *GFAP)* were upregulated in the NPC organoids after VPA treatment. VPA also upregulated several genes involved in autophagic flux, including genes involved in autophagosome–lysosome fusion *(VMAP5, SNAP25*, and *BECLIN2)* and cholesterol transport. These results suggest that in NPC organoids treated with VPA, neuronal differentiation is rescued and autophagic flux and cholesterol homeostasis are restored.

In this study, we present the first NPC brain organoid model, which provides a new method for studying the pathophysiological mechanisms involved in the neural development of NPC. Compared to the 2D culture system, this new model can provide a deeper understanding of the alterations in neuronal differentiation, proliferation, and apoptosis, including alterations in autophagy, in the 3D environment system. Furthermore, the possible roles of this model in the effective screening of drugs and clinical treatments for NPC patients were verified.

## Supplementary information

Supplementary Table 1

Supplementary figure legend

Supplementary figure 1

Supplementary figure 2

Supplementary figure 3

Supplementary figure 4

Supplementary figure 5
