# Supplementary material for: Human iNSC-derived brain organoid model of lysosomal storage disorder in Niemann–Pick disease type C
Source: Cell Death Dis. 2020 Dec 12;11(12):1059. doi: 10.1038/s41419-020-03262-7 (PMC7733597; doi:10.1038/s41419-020-03262-7)
Supplement: Supplementary file 1 — Supplementary Table 1 [file 41419_2020_3262_MOESM1_ESM.docx]

| Gene | Primer(forward) | Primer(reverse) |
| --- | --- | --- |
| *SOX2* | TGG CGA ACC ATC TCT GTG GT | CCA ACG GTG TCA ACC TGC AT |
| *PAX6* | CCA GGG CAA TCG GTG GTA G | ATC GTT GGT ACA GAC CCC CT |
| *TBR2* | TCC TTT CAC CCC AAC AGA GC | TTT GTT GGT CCC AGG TTG CT |
| *TUJ1* | GAC CCC AGC GGC AAC TAC GTG | ACG TAC TTG TGA GAA GAG GCC TCG T |
| *MAP2* | CAG GTG GCG GAC GTG TGA AAA TTG AGA GTC | CAC GCT GGA TCT GCC TGG GGA CTG TG |
| *NEUROFILAMENT* | GGC ACT GAA AAG CAC CAA GG | TGT GAG TGG ACA CAG AGG GA |
