# Supplementary material for: Human iNSC-derived brain organoid model of lysosomal storage disorder in Niemann–Pick disease type C
Source: Cell Death Dis. 2020 Dec 12;11(12):1059. doi: 10.1038/s41419-020-03262-7 (PMC7733597; doi:10.1038/s41419-020-03262-7)
Supplement: Supplementary file 2 — Supplementary figure legend [file 41419_2020_3262_MOESM2_ESM.docx]

**­Supplementary figure and table legends**

**Supplementary fig1. Generation of WT and NPC brain organoids**

(a) Bright-field images of WT organoids and NPC organoids at day 3, day 6 and day 28. (b) Quantification of diameter (mm) of WT and NPC organoids at days 28. Scale bars=500 μm. Results are mean ±SD. **p<0.01, ***p<0.001. (n=15 per sample)

**Supplementary fig2. Downregulated neuronal markers in NPC brain organoids**

(a) Immunostaining for neuronal markers (TUJ1, NF, and MAP2) in the WT and NPC organoids at day 28. Scale bars, 50μm. (b) Quantification of neuronal markers of each neuronal marker. Results are mean ±SD. **p<0.01, ***p<0.001.

**Supplementary fig3. Cholesterol accumulation in lysosome**

(a) Z-stack confocal imaging with co-localization of cholesterol (filipin staining) and lysosome (LAMP1) in the WT and NPC organoids at day 28. Scale bars, 20μm.

**Supplementary fig4. Upregulated neuronal markers in HPBCD-, VPA-treated NPC organoids**

Immunostaining for TUJ1(a), NF(c) in the non-treated NPC organoids, VPA-, and HPBCD treated NPC organoids on day 28. Quantification of neuronal markers of each neuronal marker. TUJ1(b), NF(d). Scale bars, 50μm. Results are mean ±SD. *p<0.05, **p<0.01, and ***p<0.001.

**Supplementary fig5. RNA-seq analysis of NPC organoids and VPA-treated NPC organoids.**

(a, b) Venn diagram showing the top 5 biological process and molecular function using the top 1000 upregulated genes in VPA-treated NPC organoids. (c) Heat map of differentially expressed genes of neuronal category in WT, NPC and VPA-treated NPC organoids.

**Supplementary Table 1. Primers used for qPCR. Related to Figure 1**
