# Supplementary figures and images for: Human iNSC-derived brain organoid model of lysosomal storage disorder in Niemann–Pick disease type C
Source: Cell Death Dis. 2020 Dec 12;11(12):1059. doi: 10.1038/s41419-020-03262-7 (PMC7733597; doi:10.1038/s41419-020-03262-7)

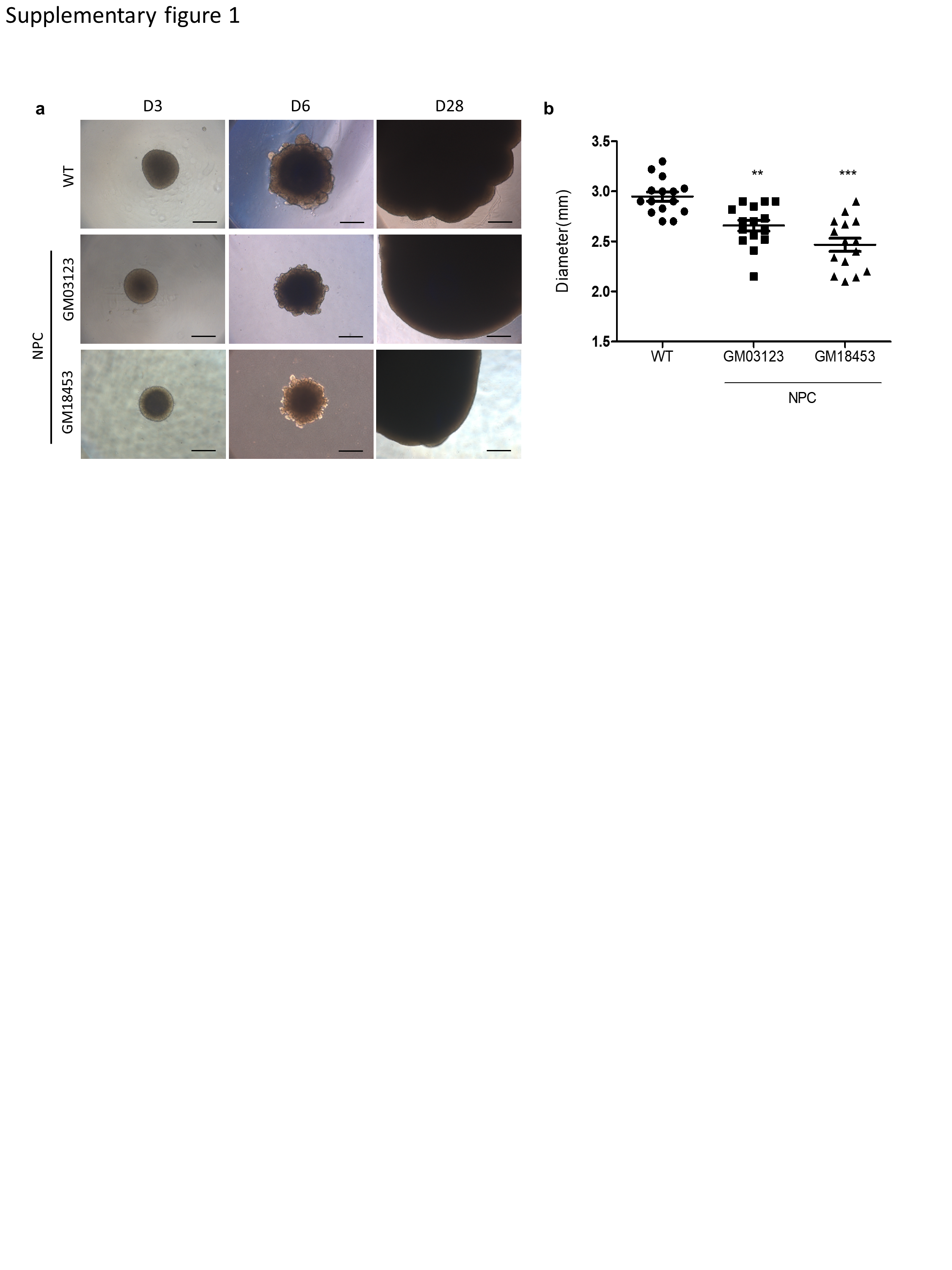

Supplement: Supplementary file 3 — Supplementary figure 1 [file 41419_2020_3262_MOESM3_ESM.tif]

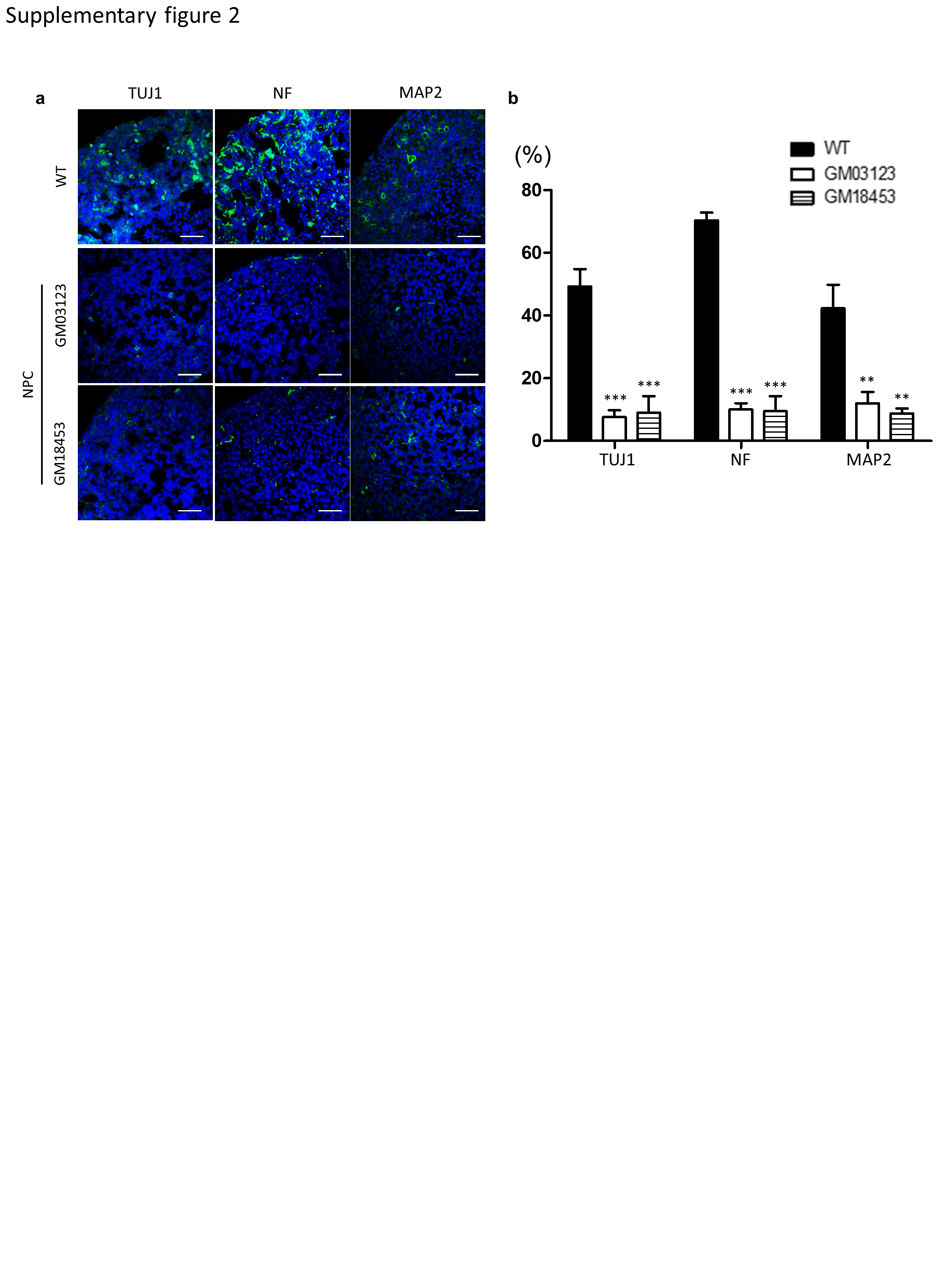

Supplement: Supplementary file 4 — Supplementary figure 2 [file 41419_2020_3262_MOESM4_ESM.tif]

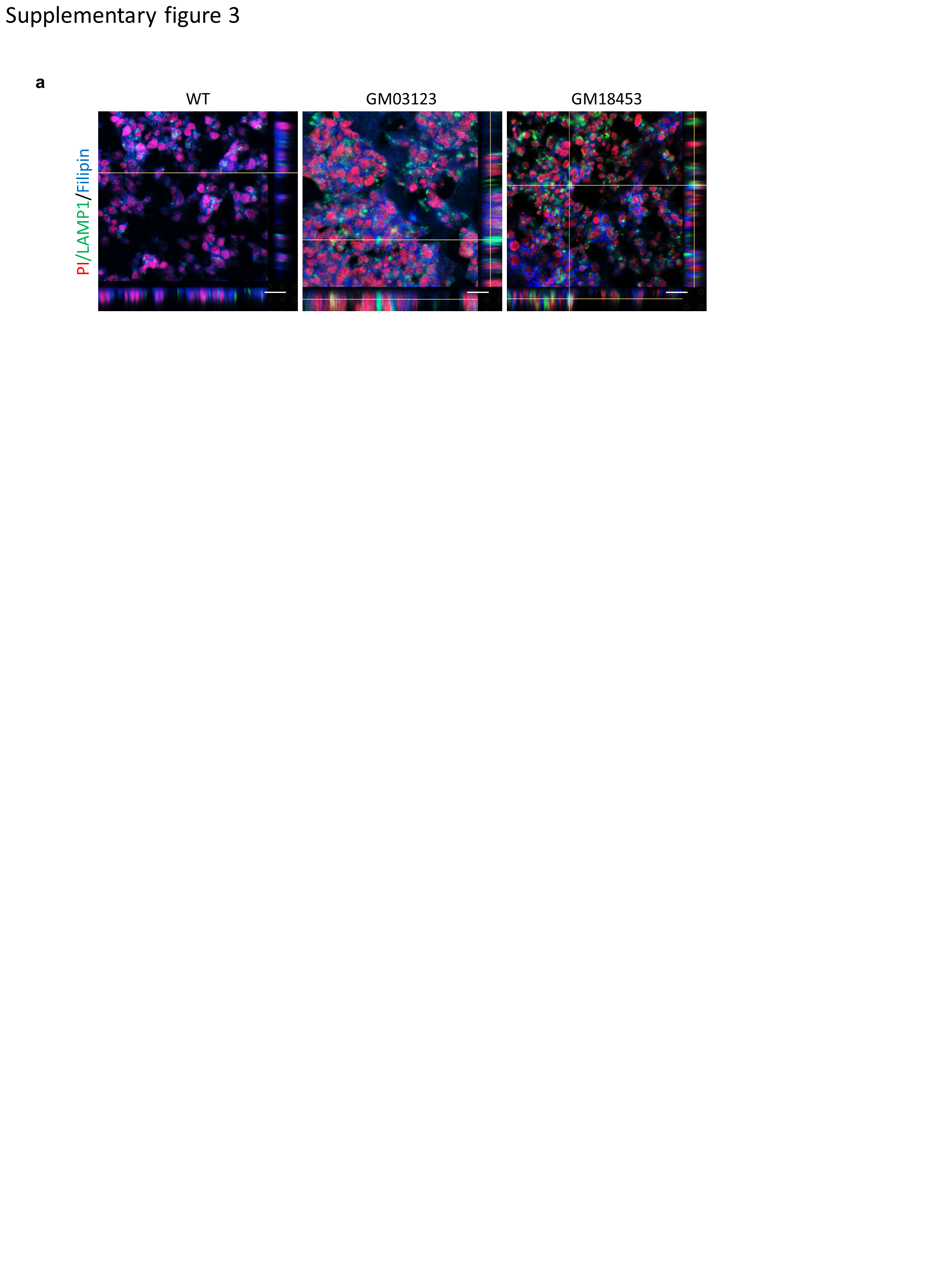

Supplement: Supplementary file 5 — Supplementary figure 3 [file 41419_2020_3262_MOESM5_ESM.tif]

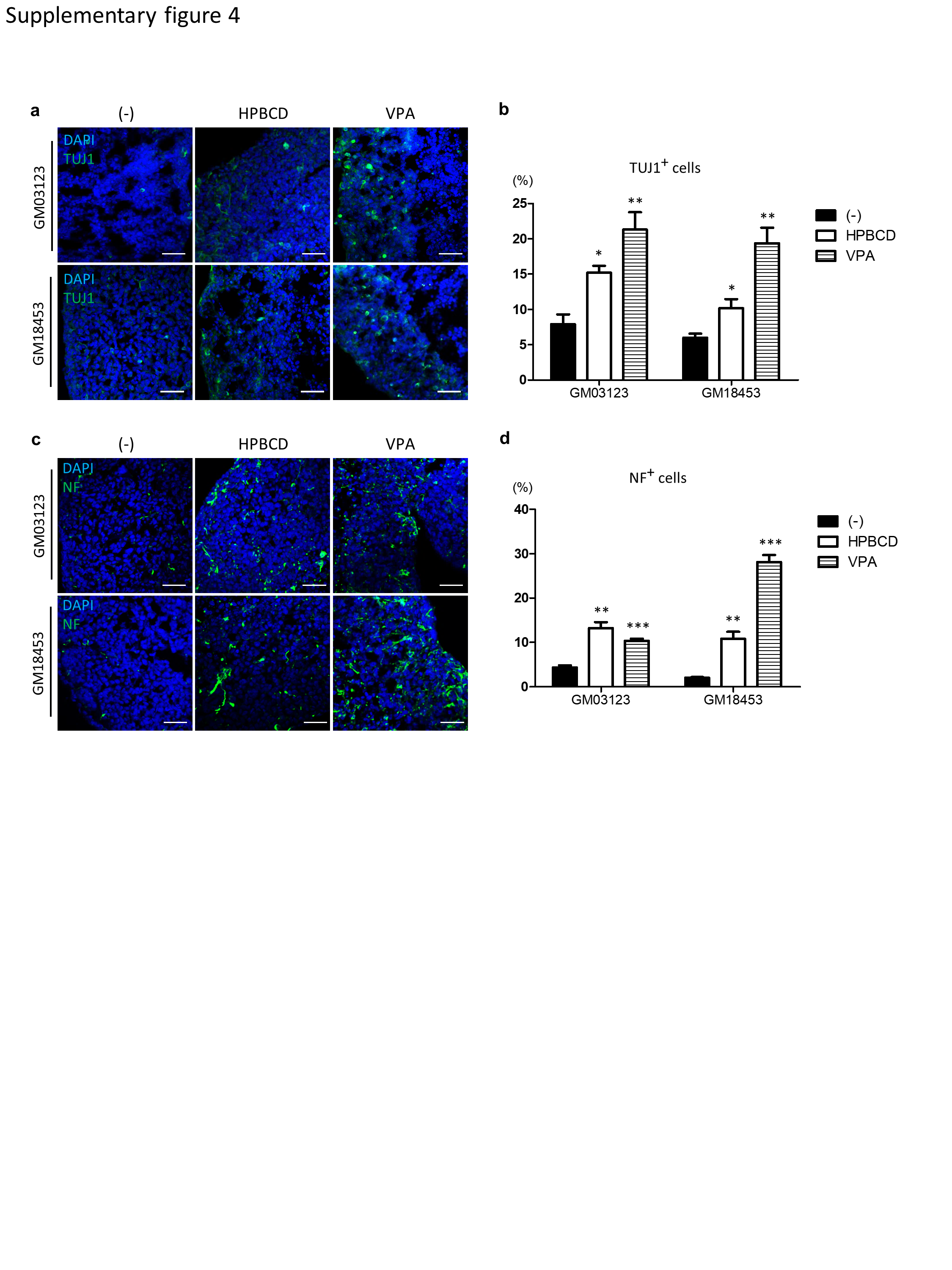

Supplement: Supplementary file 6 — Supplementary figure 4 [file 41419_2020_3262_MOESM6_ESM.tif]

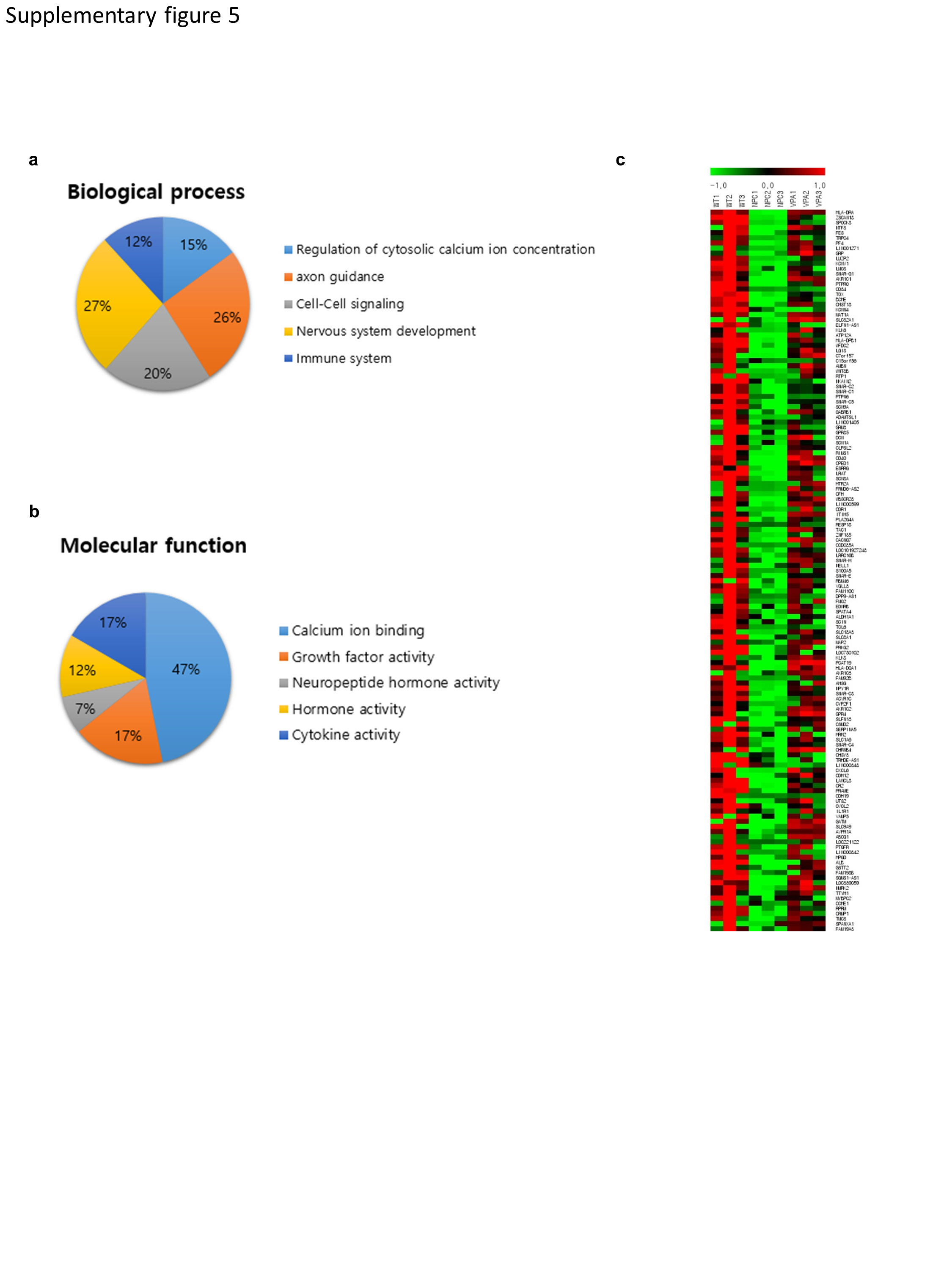

Supplement: Supplementary file 7 — Supplementary figure 5 [file 41419_2020_3262_MOESM7_ESM.tif]
